# Endogenous Peptides Identified in Soy Sauce Aroma Style Baijiu Which Interacts with the Main Flavor Compounds during the Distillation Process

**DOI:** 10.3390/foods11213339

**Published:** 2022-10-24

**Authors:** Xu Zhang, Xinshe Li, Yunhao Zhao, Qiang Wu, Yong Wan, Yougui Yu

**Affiliations:** 1 College of Food and Chemical Engineering, Shaoyang University, Shaoyang 422000, China; 2 Hunan Province Key Laboratory of New Technology and Application of Ecological Baijiu Production, Shaoyang University, Shaoyang 422000, China

**Keywords:** soy sauce aroma style baijiu, endogenous peptide, flavor substance, correlation, molecular docking

## Abstract

Endogenous peptides in Chinese baijiu have been recently reported. However, little information is available on their correlation with the main flavor substances. One hundred and forty-six peptides, consisting of more bitter amino acids and key amino acids responsible for bioactivity, were identified in tail liquor using liquid chromatography-mass spectrometry/mass spectrometry (LC-MS/MS). Additionally, the content of endogenous peptides increased gradually with distillation time, showing a high negative correlation with total esters (*r* = −0.929) and total alcohol (*r* = −0.964) but presented a moderate positive correlation with the total acid content (*r* = 0.714). The results of the correlation analysis between them were further proved by molecular docking, which showed that these endogenous peptides in baijiu interacted with the main flavor substances via hydrogen bonds. This study clarifies the dynamic changes of endogenous peptides during distillation and provides a theoretical reference for the relationship between these peptides and the main flavor substances.

## 1. Introduction

Chinese baijiu is a traditional fermented product produced by the distillation of protein-rich fermented grains such as sorghum, corn, wheat, glutinous rice, and regular rice [1]. Chinese baijiu can be divided into 12 types of flavor liquors, one of them being soy sauce aroma style baijiu. The brewing process of soy sauce aroma style baijiu is “12987”, which specifically refers to 1 year of fermentation, two times of feeding, nine times of cooking, followed by eight times of fermentation, and seven times of collecting liquors. Recent research on soy sauce aroma style baijiu has concentrated on fermentation technology and the identification of volatile and non-volatile flavor substances. However, research on the formation mechanism of these flavor substances is not available. Non-volatile substances reported in liquor mainly include pyrazines, advanced fatty acid esters, polyols, and minerals [2], and endogenous peptides are rarely identified. Moreover, endogenous peptides derived from baijiu have a variety of metabolic and physiological regulatory functions, including immunity, hormone regulation, antibacterial, antiviral, blood pressure lowering, and blood lipid regulation [3], and possess an apparent advantage of easier digestion and absorption than other flavor substances.

Moreover, nonvolatile peptides present in liquor can interact with volatile flavor compounds and change the distribution coefficient of volatile flavor compounds to improve flavor characteristics. Scalone et al. verified the effect of whey protein hydrolysate on the generation of flavor volatiles using headspace-solid phase microextraction-gas chromatography-mass spectrometry (HS-SPME-GC/MS). The presence of oligopeptides derived from whey protein hydrolysate played a major role in the generation of pyrazines. However, free amino acids contributed to a lesser extent to pyrazine formation [4]. It was found that the tetrapeptide Asp-Arg-Ala-Arg (DRAR) identified from Jingzhi Zhima aroma-type baijiu had an inhibition rate ranging from 0.09% to 39.2% of the volatilization of aromatic compounds and showed better inhibitory effects on esters and alcohols because of their varied intermolecular interactions [5]. Additionally, Ala-Lys-Arg-Ala (AKRA) from Jingzhi Zhima aroma-type baijiu interacts with p-cresol via hydrogen bonds to form the AKRA-p-cresol complex, thereby reducing the headspace concentration of phenolic off-flavors [6]. It was found that cyclooctapeptide lichenysin in Chinese strong-aroma liquor selectively affected the volatility of aromatic substances in liquor [7]. Cys-Trp-Cys (CWC) from Guojing baijiu displayed strong antioxidant and scavenging activities against intracellular reactive oxygen species [8]. We also found a novel tetrapeptide from distilled spent grains of Chinese strong-flavor baijiu, which exerted vasodilation in Ang I-stimulated endothelial cells via eNOS and END1 pathways [9]. Furthermore, the physiological activity and formation mechanism of these endogenous peptides in baijiu are important scientific issues that need to be elucidated. However, there are few studies on the dynamic alteration and trend of endogenous peptides in baijiu to clarify their formation mechanism during the distillation process, which can contribute to increasing the peptide concentration in subsequent liquor-receiving processes.

In this study, endogenous peptides were identified from the liquors collected during the distillation process of soy sauce aroma baijiu by liquid chromatography-mass spectrometry/mass spectrometry (LC-MS/MS). The distribution and dynamic alteration of endogenous peptides and the main flavor substances (acid, ester, and alcohol) during the distillation process were analyzed, and the correlation between them was explored using the Spearman correlation method. Molecular docking of these endogenous peptides and the main flavor substances was performed. This can provide a theoretical basis for extracting liquor during distillation to improve the content of endogenous peptides in soy sauce aroma-style baijiu, which is conducive to the extraction and classification of liquors to improve the quality of baijiu.

## 2. Material and methods

### 2.1. Chemicals

Methanol and acetonitrile (99% purity) were purchased from Aladdin Company (Shanghai, China). All standards used in this study, including lactic acid, propanoic acid, hexanoic acid, ethanol, methanol, pentanol, *n*-propanol, ethyl lactate, ethyl acetate, and ethyl formate, were purchased from Sigma-Aldrich (St. Louis, MO, USA). All other chemicals and reagents used in this study were of analytical grade and were obtained from the Sinopharm Chemical Reagent Company (Beijing, China).

### 2.2. Collection of Liquors during the Distillation Process

The liquors were collected at different distillation times and stages of soy sauce aroma-style baijiu production (Xiangjiao Brewing Company, Shaoyang, Hunan, China). The total production was 40 L per distillation process, based on the actual production in the Xiangjiao Brewing Company (Shaoyang, Hunan, China). Liquors (500 mL) were collected at different distillation times (5, 10, 15, 20, 30, 40, and 50 min). In addition, the liquors collected at different distillation stages are expressed as head liquor, heart liquor, and tail liquor. The head liquor was distilled with a distillation time of 5 min. The ethanol content in the head liquor was 63.5% (*v*/*v*), and the yield was 2.5% (*v*/*v*). The heart liquor was distilled from 5 to 20 min and had an ethanol content of 54.2% (*v*/*v*) and a yield of 85% (*v*/*v*). The tail liquor was distilled and collected from 20 to 50 min. It had an ethanol content of 15.4% (*v*/*v*) and a yield of 12.5% (*v*/*v*). Each sampling was repeated thrice, and the liquors were sealed at 4 °C until further analysis.

### 2.3. Pretreatment of the Collected Liquors

The collected liquors were ultrafiltrated using a 10 kDa ultrafiltration membrane (15 mL, Millipore, Billerica, MA, USA) according to the manufacturer’s instructions. These filtrates were loaded onto an SPE Cartridge C18 column (7 mm I.D., 3 mL, Sigma-Aldrich, St. Louis, MO, USA), which was pre-equilibrated using 40 mL of 10% acetonitrile under normal pressure. After washing with 5 mL of 10% acetonitrile, the peptide fractions were eluted with 5 mL of 70% acetonitrile. The eluents were evaporated in a vacuum concentration system to remove acetonitrile and further lyophilized using a vacuum centrifugation concentrator (Thermo Fisher Scientific, Waltham, MA, USA).

### 2.4. Identification of Endogenous Peptides in Soy Sauce Aroma Style Baijiu

The collected fractions were re-dissolved in 40 µL of 0.1% trifluoroacetic acid solution, and the sequences of endogenous peptides in baijiu were further identified by liquid chromatography-tandem quadrupole mass spectrometry (LC-MS/MS) [10]. This was performed on a Q Exactive mass spectrometer coupled to an Easy nLC (Thermo Fisher Scientific, Waltham, MA, USA). MS data were acquired using a data-dependent top 10 method, dynamically choosing the most abundant precursor ions from the survey scan (300–1800 *m*/*z*) for HCD fragmentation. The target value was determined based on predictive automatic gain control (PAGC). The dynamic exclusion duration was 20 s. Survey scans were acquired at a resolution of 70,000 when *m*/*z* was 200, and the resolution for the HCD spectra was set to 17,500 when *m*/*z* was 200. The normalized collision energy was 27 eV, and the underfill ratio, which specifies the minimum percentage of the target value likely to be reached at the maximum fill time, was defined as 0.1%. The instrument was run in peptide recognition mode, as described in previous reports [11].

### 2.5. Isolation of Endogenous Peptides from Soy Sauce Aroma Style Baijiu

The collected fractions were loaded using an automatic sampler into a Zorbax 300SB-C18 peptide trap (15 cm long, 75 μm inner diameter, Agilent Technologies, Wilmington, DE, USA) and then separated by a liquid chromatography column (75 µm × 150 mm, RP-C18, Column Technology Inc., Fremont, CA, USA) at a flow rate of 250 nL/min. The elution conditions were set as follows: 0–50 min, the linear gradient of liquid B was from 4% to 50%; 50–54 min, the linear gradient of liquid B was from 50% to 100%; and 54–60 min, the gradient of liquid B was maintained at 100%. Liquid A was a 0.1% formic acid aqueous solution, and liquid B was a 0.1% formic acid aqueous solution containing 84% acetonitrile. Elution was performed at 25 °C, and the absorbance was determined at 220 nm using an ultraviolet detector.

### 2.6. Full-Wavelength Scanning

Full-wavelength scanning was performed using a UV/Vis spectrophotometer (UV-2700; Shimadzu, Nakagyo-ku, Kyoto, Japan). An ethanol/water solution (50% *v*/*v*) was used to dissolve the standards, including lactic acid, propanoic acid, hexanoic acid, ethanol, methanol, pentanol, *n*-propanol, ethyl lactate, ethyl acetate, and ethyl formate, at a concentration of 10 µL/mL. Full-wavelength scanning of the standard solutions and collected samples was performed with a wavelength scanning range of 185–1400 nm. Samples (750 µL) were aspirated into a 5 mm diameter cuvette. After full-wavelength scanning, the maximum absorption values of the characteristic peaks (185–200 nm, 200–220 nm, 220–250 nm, and 250–300 nm) corresponding to the acid, ester, and peptide were processed for correlation analysis.

### 2.7. Determination of the Main Chemical Indexes

The collected liquors were concentrated at 60 °C using a rotary evaporator under a low vacuum (0.09 MPa). The peptide concentration in these liquors was determined using the Coomassie brilliant blue staining method [12]. In brief, 500 µL of liquor was added to 2.5 mL of Coomassie Brilliant Blue solution with a reaction time of 5 min, and the absorbance value was measured at 595 nm. The total acid content was determined by acid-base titration [13]. Two drops of phenolphthalein indicator were added to these liquors, and then the solutions were titrated to pink color using a 0.1 mol/L NaOH standard titration solution. The volume of the NaOH standard titration solution consumed was recorded to calculate the total acid content. The total ester content was quantified using acid-base titration [14]. These liquors were added respectively with 25 mL 0.1 mol/L NaOH standard titration solution and refluxed in a boiling water bath for 30 min. After cooling to ambient temperature, the liquors were titrated with a 0.1 mol/L H_2_SO_4_ standard titration solution to a reddish color, which was recorded to calculate the total ester content. The alcohol content of the liquors was assayed using the alcohol meter method [15]. The alcohol content of the liquors at different distillation times and stages was determined using an electronic liquid densitometer (BHOM-SJ04, Shijiazhuang Baiheng General Instrument Manufacturing Co., Ltd., Shijiazhuang, Hebei, China). After the determination of the main chemical indices, the concentrations corresponding to the acid, ester, and peptide were processed for correlation analysis.

### 2.8. Molecular Docking Simulation

The identified peptides, Ser-Thr-Leu-Val-Gly-His-Asp-Thr-Phe-Thr-Lys (STLVGHDTFTK), Thr-Arg-Pro-Pro-Arg-Glu-Glu-Glu-Glu-Leu-Arg (TRPPREEELR), and Thr-Arg-Gln-Val-Glu-Glu-Arg-Val-Trp (TRQVEERVW) derived from the head liquor, heart liquor, and tail liquor, respectively, were selected for molecular docking with the main flavor substances such as ethanol, ethyl acetate, lactic acid, and 2,3,5,6-tetramethylpyrazine, based on the method described in our previous study [16]. In brief, the 3D structure of the peptides and the main flavor substances were drawn using ChemBioDraw (OriginLab Co., CambridgeSoft, Cambridge, Cambridgeshire, Britain) and converted into a PDB file. AutoDock Tools, AutoGrid, and AutoDock modules in the AutoDock software were used to simulate molecular docking between the peptides and the main flavor substances.

### 2.9. Statistical Analysis

All data were statistically analyzed using Statistical Product and Service Solutions software (SPSS, version 21.0, IBM, Armonk, NY, USA). Significant differences (*p* < 0.05) were determined using the least significant difference (LSD) range test. Correlation analysis was performed using the Spearman correlation coefficient to identify the correlation between peptide accumulation and volatilization of the main flavor substances in the liquors (head liquor, heart liquor, and tail liquor) collected at different distillation stages using the SPSS software.

## 3. Results and Discussion

### 3.1. Structural Characterization of Endogenous Peptides in Soy Sauce Aroma Style Baijiu

**Figure 1:**
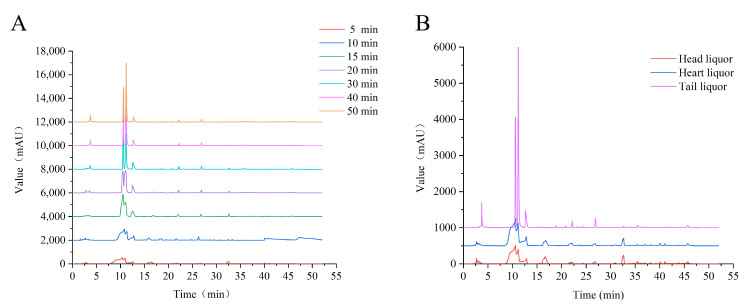
Isolation chromatograms of the peptides in liquors using RP-HPLC. (**A**) Chromatogram of the liquors collected at different distillation times. (**B**) Chromatogram of the liquors collected at different distillation stages.

**Figure 2:**
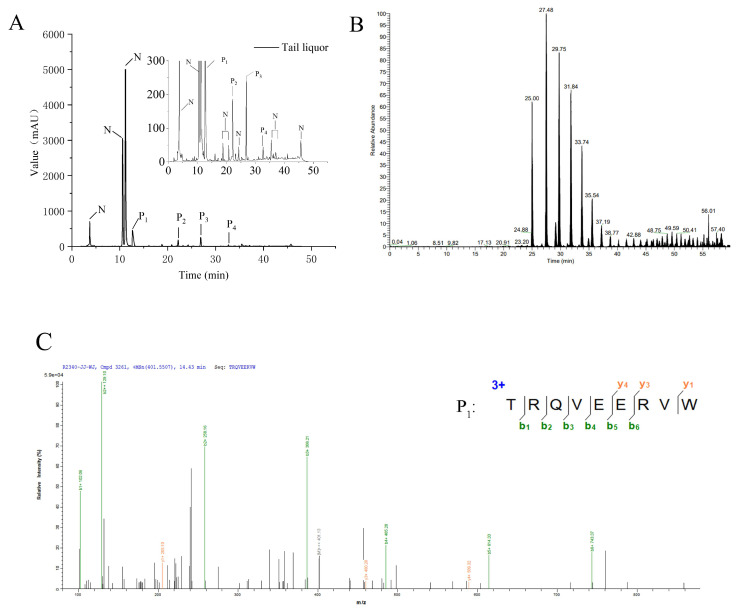

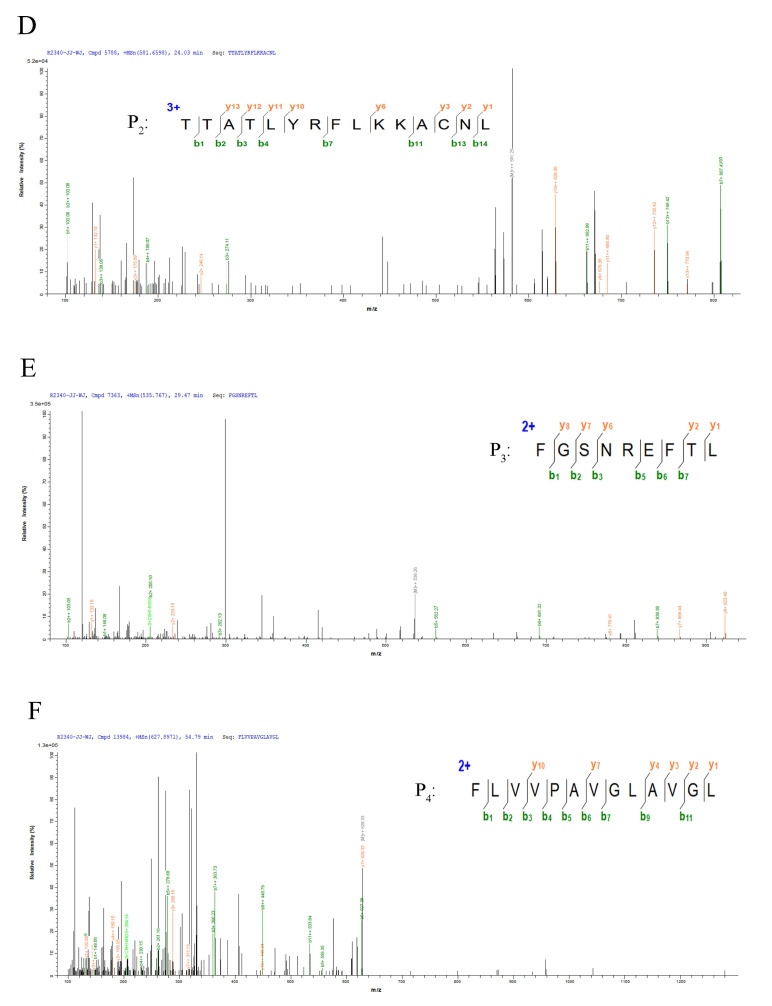
Endogenous peptides identified in tail liquor by LC-MS/MS. (**A**) The corresponding liquid chromatogram. N: no peptides. P: peptides. (**B**) Total ion chromatogram. (**C**) Secondary mass spectrogram of the peptide corresponding to the P_1_ peak. (**D**) Secondary mass spectrogram of the peptide corresponding to the P_2_ peak. (**E**) Secondary mass spectrometry of the peptide corresponding to the P_3_ peak. (**F**) Secondary mass spectrometry of the peptide corresponding to the P_4_ peak.

As shown in Figure 1, the liquors collected at an earlier distillation time (5–30 min) and in the head liquor or heart liquor presented unfavorable separation efficiency using reverse phase high-performance liquid chromatographic (RP-HPLC), suggesting complicated compositions of these liquors. Furthermore, the amino acid sequences of endogenous peptides in the head liquor, heart liquor, and tail liquor were identified using LC-MS/MS accompanied by the de novo sequencing method [17]. Under LC-MS/MS conditions, peptides are usually protonated, and the cleavage mainly occurs on amide bonds because it is difficult for other chemical bonds of side chains to break at low energy. Therefore, b and y ions are the dominant fragment ions when the collision energy is below 200 eV. Therefore, the ion fragment of the mass spectrogram obtained mainly consists of b and y ions. Ten peptides were found in the head liquor (Table S1), 16 in the heart liquor (Table S2), and 146 in the tail liquor (Table 1). The separation chromatogram and identification mass spectrogram of the head liquor are shown in Figures S1A and B, respectively. Two peptides with the amino acid sequences of Ser-Thr-Leu-Val-Gly-His-Asp-Thr-Phe-Thr-Lys (STLVGHDTFTK, RT:18.91 min, *m*/*z*:602.81; Figure S1C) and Ala-Glu-Gly-Ala-Leu-Met-Ala-Val-Gly-Asn-Ala-Glu-Ser-Arg (AEGALMAVGNAESR, RT:56.11 min, *m*/*z*:1375.67; Figure S1D) were screened according to the confidence scores (>60). Four superior peptides, including Thr-Arg-Pro-Pro-Arg-Glu-Glu-Glu-Leu-Arg (TRPPREEELR, RT:18.19 min, *m*/*z*:427.57; Figure S2C), Arg-His-Val-Arg-Pro-Gly-Thr-Val-Ala-Leu-Arg (RHVRPGTVALR, RT:23.22 min, *m*/*z*:420.59; Figure S2D), Val-Tyr-Thr-Thr-Lys-Trp-Pro-Glu-Val-Leu-Arg (VYTTKWPEVLR, RT:29.96 min, *m*/*z*:463.92; Figure S2E), and Val-Val-Tyr-Glu-Ser-Ala-Val-Gly-Asn-Ala-Glu-Ser-Arg (VVYESAVGNAESR, RT:56.16 min, *m*/*z*:1380.68; Figure S2F), were screened from the heart liquor, corresponding to P_1_, P_2_, P_3_ and P_4_ in Figure S2A, respectively. As shown in Figure 2, four peptides corresponding to the four peaks (P_1_, P_2_, P_3_ and P_4_) of the tail liquor were identified as Thr-Arg-Gln-Val-Glu-Glu-Arg-Val-Trp (TRQVEERVW, RT:14.43 min, *m*/*z*:400.88), Thr-Thr-Ala-Thr-Leu-Tyr-Arg-Phe-Leu-Lys-Lys-Ala-Cys-Asn-Leu (TTATLYRFLKKACNL, RT:24.03 min, *m*/*z*:291.81), Phe-Gly-Ser-Asn-Arg-Glu-Phe-Thr-Leu (FGSNREFTL, RT:24.03 min, *m*/*z*:291.81), and Phe-Leu-Val-Val-Pro-Ala-Val-Gly-Leu-Ala-Val-Gly-Leu (FLVVPAVGLAVGL, RT:54.79 min, *m*/*z*:627.39), respectively.

**Figure 3:**
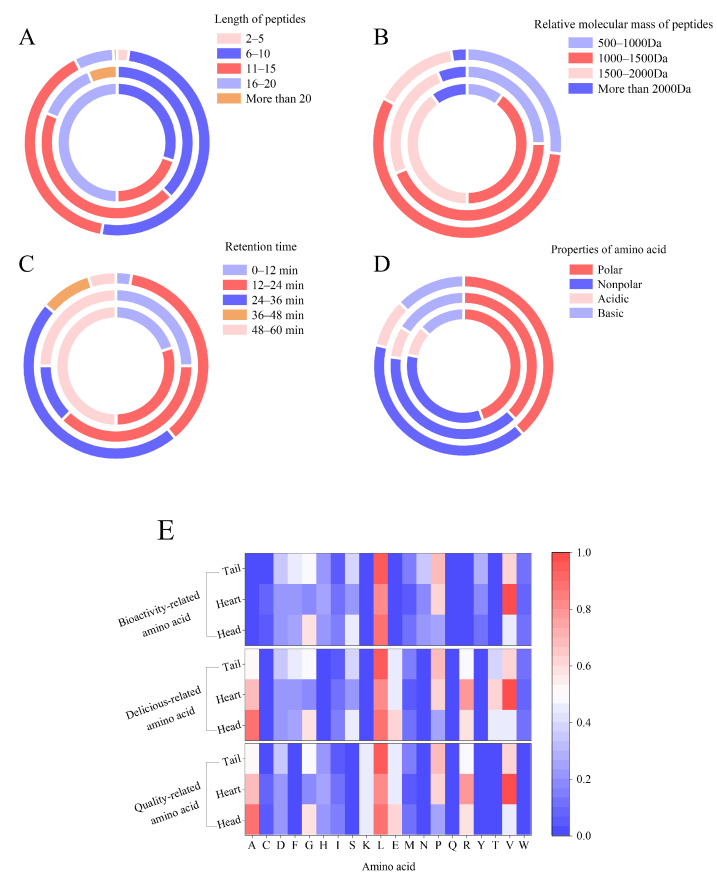
Statistic analysis of the amino acid residues occurred in peptides of the liquors from different distillation stages. (**A**) Distribution diagram of the length of the peptides. It was calculated as the proportion in the totality of amino acid residues of all peptides. The circles from inside to outside represented the head liquor, heart liquor and tail liquor. (**B**) Distribution diagram of the molecular weight of the peptides. It was calculated as the proportion in the totality of amino acid residues of all peptides. The circles from inside to outside represented the head liquor, heart liquor and tail liquor. (**C**) Distribution diagram of the retention time of the peptides. It was calculated as the proportion in the totality of amino acid residues of all peptides. The circles from inside to outside represented the head liquor, heart liquor and tail liquor. (**D**) Distribution diagram of the amino acid properties of the peptides. It was calculated as the proportion in the totality of amino acid residues of all peptides. The circles from inside to outside represented the head liquor, heart liquor and tail liquor. (**E**) Distribution diagram of amino acid of the liquors. It was calculated as the proportion in the totality of amino acid residues of all peptides. The graph from top to bottom represents the bioactive amino acid and the delicious amino acids that influenced baijiu quality.

Based on the amino acid sequences of these peptides identified in liquors collected at different distillation stages, the peptides in the head liquor presented larger molecular weight containing 16–20 amino acid residues, and the peptides in heart liquor (11–15 amino acid residues) and tail liquor (6–10 amino acid residues) later (Figure 3A). Accordingly, 80% of the molecular weights of the peptides in the head liquor were between 1000–2000 Da, and 44% and 56% of those in the heart liquor and tail liquor were between 1000–1500 Da (Figure 3B). The boiling point of peptides composed of different amino acid residues is different. Moreover, the temperature gradually increases with distillation time [18] so that more peptides are volatilized in the latter distillation time and enter the tail liquor. Based on the retention time of the separation chromatogram, 50% of the peptides in the head liquor were eluted from the C18 column at 48–60 min. However, the retention times of the peptides in the heart and tail liquor were mainly between 12–24 min and 24–36 min, respectively (Figure 3C). Moreover, as shown in Figure 3D, more polar amino acid residues were present in the peptides in the head liquor (57%), and more non-polar amino acid residues were present in the peptides in the heart liquor (51%) and tail liquor (51%). This result suggested that the non-polar amino acid residues affected the volatility of the peptides in the liquors during the distillation process because of their higher boiling point. However, there was an exception in that the alkaline amino acid residues frequently occurred in the peptides in these liquors. The bioactivity of the peptides was closely related to the composition of the amino acids, which also determines molecular weight, charge distribution, and hydrophobicity/hydrophilicity, and further affects the combination of peptides with flavor substances in the baijiu [19]. Furthermore, the key amino acids responsible for bioactivity reported are Ala, Leu, Glu, Pro, Arg, Lys, Thr, and Val [8,20,21]. In addition, some amino acids such as Ala, Gly, Leu, Glu, Pro, Arg, Thr, and Val are also associated with taste, including sweet, sour, and bitter tastes [22]. Additionally, some specific amino acids (Ala, Gly, Leu, Glu, Lys, Pro, Arg, and Val) exert a desirable influence on the quality of baijiu based on previous reports [5,6]. As shown in Figure 3E, Ala, Leu, Glu, Arg, and Gly were present in these peptides in the head liquor, occupying relatively large proportions (>40%). More amino acids, such as Val, Arg, Leu, Ala, Thr, and Pro, were observed in these peptides in the heart liquor. Leu, Pro, and Val accounted for a larger proportion of these peptides in the tail liquor. Some amino acids of the peptides in the head liquor and heart liquor were associated with sweet (Ala, Gly, and Pro), bitter (Leu, Val, Pro, and Arg), and sour tastes (Thr and Glu). However, bitter amino acids frequently occur in the peptides in the tail liquor. Ala, Leu, Glu, Arg, Lys, Thr, and Val are present in these peptides in the head liquor and account for a greater proportion. In heart liquor, a larger number of peptides were composed of Val, Leu, Lys, Thr, Pro, Lys, and Glu. However, in the tail liquor, the key amino acids corresponding to bioactive activity were Leu, Pro, Val, Ala, Phe, Lys, Glu, and Arg, which were dominant.

### 3.2. Correlation Analysis between the Peptides and the Main Flavor Substances in Liquors Collected at Different Distillation Stages

**Figure 4:**
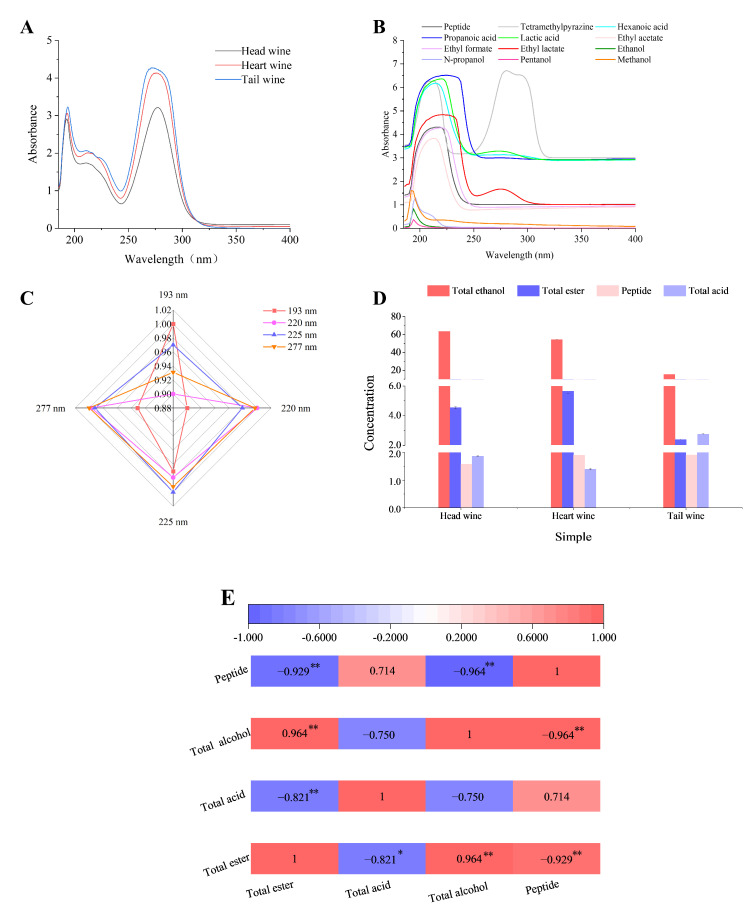
Correlation analysis between the peptides and the main flavor substances in the liquors collected at different distillation stages. (**A**) Spectra of the liquors from different distillation stages. (**B**) Spectra of the standard substances involved in baijiu. (**C**) Correlation analysis among the characteristic peaks based on the maximum absorption value. (**D**) Content determination of total acid, total ester, total alcohol and the peptide. (**E**) Correlation analysis between the content of the peptide and total acid, total ester, or total alcohol. Red represents positive, and blue represents negative. * 0.01< *p* ≤ 0.05, ** *p* ≤ 0.01.

The main constituents of baijiu are alcohols, esters, and acids [23]. The corresponding standards of the main flavor substances, including lactic acid, propanoic acid, hexanoic acid, ethanol, methanol, pentanol, *n*-propanol, ethyl lactate, ethyl acetate, and ethyl formate, were used for full-wavelength scanning in this study. As shown in Figure 4B, the alcohol (ethanol, methanol, pentanol, and n-propanol) presented a characteristic peak at 185–200 nm, which, according to a report [24], explained that the excitation of lone-pair electrons on an auxochrome as halogens, OH, O, SH, S, and NH2, respectively, to σ* or π* orbitals require less energy than the excitation of bonding σ or π electrons and the absorption shifts toward longer wavelengths. In addition to simple alcohols such as methyl alcohol (λ_max_ 183.1 nm) and ethyl alcohol (λ_max_ 181.8 nm), the absorption maxima of nonaromatic primary alcohols are below 167 nm. The acid and 2,3,5,6-tetramethylpyrazine presented different characteristic peaks at 200–220 nm, 220–250 nm, and 250–300 nm, respectively [25,26,27]. Moreover, the peak at 200–220 nm was reported as the wavelength of the peptide substance [28]. The ester showed a characteristic peak at 250–300 nm, which was consistent with the result that the compounds with terminal hydroxyl group absorbed with maxima around 280–290 nm, while the derivatives with cyanate and carbamate extremities presented broad bands around 250–275 nm [29].

It presented an obvious difference in the wavelengths of 185–200 nm, 200–220 nm, 220–250 nm, and 250–300 nm of the liquors collected at different distillation stages by full-wavelength scanning (Figure 4A). As shown in Figure 4C, the correlation analysis results showed the following: the maximum absorption value at 193 nm was positively correlated with the maximum absorption value at 220 nm (*r* = 0.906), 225 nm (*r* = 0.970), and 277 nm (*r* = 0.931); the maximum absorption value at 193 nm was positively correlated with the maximum absorption value at 225 nm (*r* = 0.750); the maximum absorption value at 225 nm was positively correlated with the maximum absorption values at 220 nm (*r* = 0.982) and 277 nm (*r* = 0.992); and there was a significant correlation between the maximum absorption value at 220 nm and the maximum absorption value at 277 nm (*r* = 0.998, *p* < 0.05). The peptide, total acid, total ester, and total alcohol contents in the head liquor, heart liquor, and tail liquor were detected, and their correlations were analyzed. According to Figure 4D, in head liquor, heart liquor, and tail liquor, the total ester content increased from 4.55 g/L to 5.67 g/L and then decreased to 2.39 g/L. The total acid content decreased from 1.87 g/L to 1.40 g/L and then increased to 2.75 g/L. Alcohol content decreased from 63.5% (*v*/*v*) to 15.4% (*v*/*v*), while the peptide concentration increased slightly from 1.58 mg/mL to 1.91 mg/mL. As shown in Figure 4E, total ester negatively correlated with total acid content (*r* = −1.000, *p* < 0.01). The contents of total ester and total alcohol were strongly positively correlated (*r* = 0.866). There was a significant negative correlation between total acid content and total alcohol content (*r* = −0.862). There was a negative correlation between peptide content and alcohol content (*r* = −0.657). The peptide content correlated slightly with total acid content (*r* = 0.183) and total ester content (*r* = −0.192).

### 3.3. Correlation Analysis between the Peptides and the Main Flavor Substances in Liquors Collected at Different Distillation Times

The collected liquors mainly peaked at wavelengths of 185–200 nm, 200–220 nm, 220–250 nm, and 250–300 nm, and their absorbance values were significantly different at different distillation times (Figure S3A). Furthermore, the Spearman correlation was used to analyze the correlation among these characteristic peaks at 185–200 nm, 200–220 nm, 220–250 nm, and 250–300 nm (Figure S3B). The results showed that the absorbance value at 193 nm corresponding to the acids (lactic acid, propanoic acid, and hexanoic acid) was significantly positively correlated with the absorbance value at 220 nm (the peptide) and 277 nm corresponding to the esters (ethyl lactate, ethyl acetate, and ethyl formate), respectively, with correlation coefficients of *r* = 0.786 (*p* <0.05) and *r* = 0.893 (*p* < 0.01). There was a positive correlation between the absorbance values at 193 and 225 nm (*r* = 0.750). The absorbance at 220 nm showed a significant positive correlation with the absorbance at 225 nm (*r* = 0.964, *p* < 0.01) and 277 nm (*r* = 0.964, *p* < 0.01). Moreover, the absorbance values at 225 and 277 nm showed a significant positive correlation (*r* = 0.929, *p* < 0.01).

As shown in Figure S3C, at the earlier distillation time (0–30 min), the total acid content in the collected liquors showed an overall upward trend and reached a maximum at 3.53 g/L, while between 30–50 min of distillation time, the total acid content decreased from 3.53 g/L to 3.15 g/L. During the whole distillation process, the content of the total esters decreased from 5.95 g/L to 1.95 g/L, and the content of total alcohol decreased from 53.9% (*v*/*v*) to 1.9% (*v*/*v*). On the contrary, the content of total peptides increased slightly from 1.58 mg/mL to 1.85 mg/mL. As exhibited in Figure S3D, correlation analysis showed that there was a significant negative correlation between total acid and total ester content (*r* = −0.820, *p* < 0.05) and a significantly positive correlation between the total esters and total alcohol content (*r* = 0.964, *p* < 0.01). Total acid and alcohol levels showed a significant negative correlation (*r* = −0.750). The correlation analysis results of the liquors collected at different distillation stages were consistent with the results of different distillation times (Figure 4). This might be because esters are difficult to dissolve in water and easily dissolve in ethanol and other organic solvents. Ethanol is continuously distilled in the early stage, while the esters dissolve in ethanol and flow out together with the distillate [30]. In addition, acid compounds are mostly concentrated in late distillations because most organic acids are easily soluble in water but hardly soluble in ethanol, and their boiling points are high [31]. At the later stage of distillation, ethanol in the fermented grains was completely distilled, and more water was distilled out, so the total alcohol content showed a downward trend. The peptide content detected in the collected liquors gradually showed an increasing trend during the distillation process, which may be because peptides are hydrophilic substances with high boiling points and are carried out with distilled water in the tail liquor [2]. There was a significant negative correlation between the peptide content, total ester content, and total alcohol content detected at different distillation times, with correlation coefficients of *r* = −0.929 (*p* < 0.01) and *r* = −0.964 (*p* < 0.01). There was a positive correlation between peptide and total acid levels (*r* = 0.714). It was confirmed that the ester and acid contain carboxyl groups, and the alcohol contains hydroxyl groups, which can form hydrogen bonds with amide bonds or other chemical bonds in peptides, affecting the volatility and viscosity of volatile compounds [32].

### 3.4. Molecular Docking between the Typical Peptides and the Main Flavor Substances in Liquors Collected at Different Distillation Stages

STLVGHDTFTK in head liquor, TRPPREEELR in heart liquor, and TRQVEERVW in tail liquor were selected for molecular docking simulations with ethanol, ethyl acetate, lactic acid, and 2,3,5,6-tetramethylpyrazine, respectively (Table 2). As shown in Figure S4A, His and Asp in STLVGHDTFTK from the head liquor form two hydrogen bonds with the hydroxyl group of ethanol. The distance of the hydrogen bonds was 2.06 Å and 2.16 Å, respectively, and the binding energy of the hydrogen bonds was 2.07 kcal/mol. His in STLVGHDTFTK formed a hydrogen bond with the carbonyl group of ethyl acetate; the hydrogen bond was 2.12 Å, and the binding energy of the hydrogen bond was 2.62 kcal/mol. The Thr in STLVGHDTFTK formed a hydrogen bond with the carboxyl group of lactic acid, the distance of the hydrogen bond was 1.87 Å, and the binding energy of the hydrogen bond was 2.84 kcal/mol. However, STLVGHDTFTK failed to form any chemical bond with 2,3,5,6-tetramethylpyrazine. As shown in Figure S4B, Glu and Arg in TRPPREEELR derived from heart liquor form two hydrogen bonds with the hydroxyl group of ethanol. The distances of hydrogen bonds were 2.04 Å and 1.84 Å, and the hydrogen-bond binding energy was 1.95 kcal/mol. Glu in TRPPREEELR formed a hydrogen bond with the carbonyl group of ethyl acetate, the distance of the hydrogen bond was 1.92 Å, and the binding energy of the hydrogen bond was 2.18 kcal/mol. Thr in TRPPREEELR formed a hydrogen bond with the carboxyl of lactic acid; the hydrogen bond was 1.97 Å, and the binding energy of the hydrogen bond was 2.45 kcal/mol. The hydrogen bond distance between Arg in TRPPREEELR and 2,3,5,6-tetramethylpyrazine was 1.66 Å, and the binding energy of the hydrogen bond was 3.90 kcal/mol. According to Figure S4C. Glu in TRQVEERVW in the tail liquor forms a hydrogen bond with the hydroxyl group of ethanol. The hydrogen bond was 2.00 Å, and the binding energy of the hydrogen bond was 2.64 kcal/mol. It also formed a hydrogen bond with the carbonyl group of ethyl acetate, the distance of the hydrogen bond was 2.79 Å, and the binding energy of the hydrogen bond was 3.53 kcal/mol. Additionally, it forms a hydrogen bond with the carboxyl group of lactic acid. The hydrogen bond was 1.87 Å, and the binding energy of the hydrogen bond was 3.32 kcal/mol. The hydrogen bond distance between Glu in TRPPREEELR and 2,3,5,6-tetramethylpyrazine was 2.54 Å, and the binding energy of the hydrogen bond was 4.65 kcal/mol. These results showed that endogenous peptides could interact with the main flavor substances, including alcohol, acid, ester, and pyrazine, via hydrogen bonding to promote or inhibit volatilization, thereby affecting the flavor of the liquor. In addition, research on the negative impact of these endogenous peptides on the volatility of flavor substances in liquor should be further studied in the future.

## 4. Conclusions

Ten peptides in the head liquor, 16 peptides in the heart liquor, and 146 peptides in the tail liquor were identified from soy sauce aroma baijiu. The production of endogenous peptides increased gradually with distillation time, showing a high negative correlation with the total ester (*r* = −0.929) and total alcohol content (*r* = −0.964) and a moderate positive correlation with total acid content (*r* = 0.714). In addition, there were obvious differences in the endogenous peptides in the liquors collected at different distillation stages. Compared with head liquor and heart liquor, there were more amino acids responsible for the bioactive activity, and bitter amino acids were present in the peptides in the tail liquor. Furthermore, molecular docking results proved that peptides formed hydrogen bonds with alcohols, acids, esters, and pyrazines, thereby promoting or inhibiting their volatilization, which will provide direction for future research and further explore the binding mechanism of peptides and flavor substances in baijiu.

## Figures and Tables

**Table 1:** Composition of endogenous peptide in tail liquor.

Simple	Sequence	Length	Retention Time	Mass	*m*/*z*	Sequence	Length	Retention Time	Mass	*m*/*z*
Tail liquor	YVLSKVPAPLT	12	43.45	1187.71	593.85	RLEEWVYKWQPSTALRMPRHDRDYL	8	20.26	943.49	188.70
	VYPFPGLP	13	43.70	889.48	444.74	SKRPTLSNLGYGR	12	20.35	945.49	315.16
	SVGAVWPEV	12	44.20	943.49	471.74	KAGEKELKVFGTQELLNL	10	20.85	1298.78	432.93
	SVGGMVLPW	13	44.47	945.49	945.49	EPTVDDEALEK	12	20.98	1298.77	649.39
	FRPLGKATRGVP	11	45.19	1298.78	649.39	NYPKARVSATL	8	21.00	1298.77	649.38
	PAVAFKSPVLRN	8	45.69	1298.77	649.39	ARDLPGVKLGSDL	15	21.13	1282.78	427.59
	PAVAFKSPVLRN	15	47.21	1298.77	649.38	DVGHYPYFQHLLGPA	12	21.44	1143.72	381.24
	VRTLGAAKTPVAV	9	48.39	1282.78	641.39	HRLSSDLKPGMV	9	21.45	1409.84	469.95
	LFVAVAVAVRV	11	48.81	1143.72	571.86	HEEPVAPTL	10	21.51	1320.76	660.38
	VFVRSKAGVRKY	15	49.95	1409.84	704.92	TPRSKLSDLGYGR	16	21.69	1520.92	506.97
	VPVVPPFLQPEV	8	51.59	1320.76	660.38	GLHAESTWYPAY	10	21.90	1254.79	627.39
	PPPLVQKPVGLFTK	10	53.60	1520.92	760.46	GEDAHHPLYL	11	22.02	1041.55	520.77
	FLVVPAVGLAVGL	12	54.79	1254.79	627.39	AHAALASADL	9	22.26	844.52	422.26
	VEELKPTPE	9	0.61	1041.55	520.77	RSRDPLLTVEYLGK	16	22.39	1365.85	341.46
	FLVGRPR	4	0.62	844.52	422.26	PAAEQKKYPRTKMLNL	11	22.46	1202.64	400.88
	LQPVTKAVAVGLAV	13	59.28	1365.85	682.93	PAAEQKKKMEKTLLGCL	11	22.47	1202.64	300.66
	RTQVEEKEAL	7	14.39	1202.64	601.32	GEGGTGHPV	17	22.52	1948.91	1948.91
	TRQVEERVW	9	14.43	1202.64	400.88	KEAFGVLN	12	22.73	1059.48	529.74
	LDARRQSTP	9	16.73	1043.56	521.78	KFPPYPPLPMKNMLFK	11	23.11	993.55	331.18
	RLDLAGDR	6	16.94	915.50	457.75	LNRDSSLLEVQLQNLDR	9	23.41	860.45	215.11
	GGSKRQFTL	10	17.00	993.55	496.77	RQLPAGTTYY	12	23.52	985.51	492.75
	KNKDEGGL	14	17.44	860.45	430.22	LFMQYFLRL	17	23.54	1355.71	677.85
	YNLKEYR	11	17.74	985.51	492.75	EHALFSVD	10	23.77	949.47	474.73
	GPTYKLSRDRY	8	18.05	1355.71	451.90	DVLDGNHL	15	23.84	1247.66	623.83
	DFQMPRR	10	18.07	949.47	474.73	NALMTHFVWAVPGGL	7	23.91	980.51	326.84
	KFNQYGHVVR	11	18.36	1247.66	415.89	TTATLYRFLKKACNL	9	24.03	875.44	291.81
	PVNNPHFR	7	18.47	980.51	490.25	AHAALATGDVSL	8	24.26	1305.63	652.82
	SDRFHTL	13	18.48	875.44	437.72	FKSDRLF	10	24.41	1412.70	706.35
	TEEFGEKLQQP	12	18.55	1305.63	652.82	FELMKRASTF	9	25.21	1079.50	359.83
	HENLPTLHFGTF	5	18.58	1412.70	470.90	TLEVVCFQTL	9	25.44	913.49	456.74
	LREQFEEE	11	18.89	1079.50	539.75	RAQLQWGVPFKF	8	25.45	1263.58	631.79
	LPHFNSKA	8	18.93	913.49	456.74	GRVLLVPQN	10	25.54	955.56	477.78
	DLSNADREKSE	17	18.95	1263.58	631.79	RDPLYSRLTLGF	9	25.59	1008.46	336.15
	KNPQLKDL	12	19.11	955.56	477.78	AGDDAPRAVF	15	25.61	1018.50	509.25
	GAEFVQTQE	14	19.16	1008.46	504.23	ASERVGLLHSQNTSL	11	25.88	1611.85	537.28
	KNLPQEVR	12	19.57	1187.71	593.85	FNTEVPAM	12	25.96	924.41	924.41
	SPYSPYPMR	8	19.62	889.48	444.74	RFNEFGHL	9	26.11	1019.51	509.75
	LAPVNKPYEWQF	25	26.20	1491.76	497.25	DLNFTEPR	9	26.52	991.48	495.74
	EFLTPEKNPQLDR	18	26.21	1586.82	793.41	PAAFLPSNVEKL	14	26.59	1285.72	428.57
	ALPVNKFWTTWE	20	26.23	1491.76	745.88	GAENPELTSGHS	6	26.70	1198.54	599.27
	YAHTLNHMKVGNKFAY	15	26.25	1893.95	631.32	FTLE	12	26.74	509.26	509.26
	YYWNLKGCPF	10	26.31	1290.60	645.30	THPSVADEFYR	16	26.96	1321.62	440.54
	QGKGAVVLLGL	8	26.32	1054.66	527.33	NEYLEDQL	12	27.08	1023.46	511.73
	RHVIMAVG	8	26.046	881.49	440.75	RHVIMAVG	8	26.046	881.49	440.75
	STSLTLMMLLSA	12	33.382	1266.66	633.33	STSLTLMMLLSA	12	33.382	1266.66	633.33
	STSPKRPSNSN	11	24.686	1173.57	586.79	STSPKRPSNSN	11	24.686	1173.57	586.79
	VGTDRSVILFDL	12	23.936	1333.72	666.86	VGTDRSVILFDL	12	23.936	1333.72	666.86
	VILTVRDVDD	10	0.03875	1143.61	571.81	VILTVRDVDD	10	0.03875	1143.61	571.81
	WFIDFHRT	8	44.244	1120.55	560.27	WFIDFHRT	8	44.244	1120.55	560.27
	RSRDPLYSNKLGKF	12	23.02	1043.56	260.89	GAEPFFGQQQPLTWDVE	11	16.09	1948.91	649.64
	CTLESAGLEH	10	16.48	1059.48	529.74	KFPYYLSHKFVMLKF	9	23.09	915.50	228.88

**Table 2:** Molecular docking results between the typical peptides and the major flavor substances.

Ligand	Source	Peptides	Binding Energy	Amino Acid Residues	Number of Hydrogen Bonds	Hydrogen Bond Distance
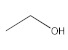	Head liquor	STLVGHDTFTK	2.07	His (HN)/Asp (O)	2	2.16/2.06
	Heart liquor	TRPPREEELR	1.95	Glu (O)/Arg (HN)	2	2.04/1.84
	Tail liquor	TRQVEERVW	2.64	Glu (O)	1	2.00
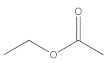	Head liquor	STLVGHDTFTK	2.62	His (HN)	1	2.12
	Heart liquor	TRPPREEELR	2.18	Glu (O)	1	1.92
	Tail liquor	TRQVEERVW	3.32	Glu (O)	1	1.87
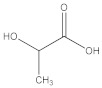	Head liquor	STLVGHDTFTK	2.84	Thr (O)	1	1.87
	Heart liquor	TRPPREEELR	2.45	Arg (HN)	1	1.97
	Tail liquor	TRQVEERVW	3.53	Glu (O)	1	2.79
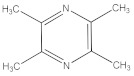	Head liquor	STLVGHDTFTK	3.88	Thr (O)	/	/
	Heart liquor	TRPPREEELR	3.90	Arg (HN)	1	1.66
	Tail liquor	TRQVEERVW	4.65	Glu (O)	1	2.54

/ represented no hydrogen bonds form.

## Data Availability

Data are contained within the article or the Supplementary Materials.

## Supplementary Materials

The following supporting information can be downloaded at: https://www.mdpi.com/article/10.3390/foods11213339/s1, Table S1: Composition of endogenous peptide in head liquor; Table S2: Composition of endogenous peptide in heart liquor; Figure S1: Endogenous peptides identified in head liquor by LC-MS/MS; Figure S2: Endogenous peptides identified in heart liquor by LC-MS/MS; Figure S3: Correlation analysis between the peptides and the main flavor substances in baijiu samples from different distillation stages; Figure S4: Molecular docking between the peptides and the major flavor substances.

## References

[1] Hong J., Tian W., Zhao D. (2020). Research progress of trace components in sesame-aroma type of baijiu. Food Res. Int..

[2] Wu J., Huang M., Zheng F., Sun J., Sun X., Li H., Sun B. (2019). Baijiu health and healthy Baijiu. J. Food Sci. Tech..

[3] Acevedo-Juárez S., Guajardo-Flores D., Heredia-Olea E., Antunes-Ricardo M. (2022). Bioactive peptides from nuts: A review. Int. J. Food Sci. Technol..

[4] Scalone G.L.L., Cucu T., De Kimpe N., De Meulenaer B. (2015). Influence of free amino acids, oligopeptides, and polypeptides on the formation of pyrazines in Maillard model systems. J. Agric. Food Chem..

[5] Huang M., Huo J., Wu J., Zhao M., Sun J., Zheng F., Li H. (2019). Structural characterization of a tetrapeptide from Sesame flavor-type Baijiu and its interactions with aroma compounds. Food Res. Int..

[6] Huang M., Huo J., Wu J., Zhao M., Zheng F., Sun J., Li H. (2018). Interactions between p-Cresol and Ala-Lys-Arg-Ala (AKRA) from sesame-flavor-type baijiu. Langmuir.

[7] Zhang R., Wu Q., Xu Y. (2014). Lichenysin, a cyclooctapeptide occurring in Chinese liquor Jiannanchun reduced the headspace concentration of phenolic off-flavors via hydrogen-bond interactions. J. Agric. Food Chem..

[8] Peng L., Kong X., Wang Z., Ai-Lati A., Ji Z., Mao J. (2021). Baijiu vinasse as a new source of bioactive peptides with antioxidant and anti-inflammatory activity. Food Chem..

[9] Wu Q., Zhong C., Zeng G., Zhang X., Xiang L., Wan C., Yu Y. (2022). Identification and characterization of a novel tetrapeptide from enzymatic hydrolysates of Baijiu byproduct. Food Sci. Hum. Well..

[10] Zhang L., Jiang Y., Yin Z., Sun J., Li H., Sun X., Zheng F. (2018). Isolation and evaluation of two angiotensin-I-converting enzyme inhibitory peptides from fermented grains (Jiupei) used in Chinese Baijiu production. RSC Adv..

[11] Zheng Q., Wang Z., Xiong A., Hu Y., Su Y., Zhao K., Yu Y. (2021). Elucidating oxidation-based flavour formation mechanism in the aging process of Chinese distilled spirits by electrochemistry and UPLC-Q-Orbitrap-MS/MS. Food Chem..

[12] Kurien B.T., Scofield R.H. (2018). Application of Heat to Quickly Stain and Destain Proteins Stained with Coomassie Blue. Protein Gel Detection and Imaging.

[13] Fetzer W.R., Jones R.C. (1952). Determination of free and total acidity in commercial lactic acid. Anal. Chem..

[14] Wang C., Zhao F., Bai Y., Li C., Xu X., Kristiansen K., Zhou G. (2022). *In vitro* digestion mimicking conditions in young and elderly reveals marked differences between profiles and potential bioactivity of peptides from meat and soy proteins. Food Res. Int..

[15] Xu C., Wu J., Liu X., Shi W. (2021). Research on the measurement of alcohol density concentration and the convenient formula. China Meas. Test.

[16] Wu Q., Li Y., Peng K., Wang X.L., Ding Z., Liu L., Xu P., Liu G.Q. (2019). Isolation and characterization of three antihypertension peptides from the mycelia of Ganoderma lucidum (Agaricomycetes). J. Agric. Food Chem..

[17] Allmer J. (2011). Algorithms for the de novo sequencing of peptides from tandem mass spectra. Expert Rev. Proteomic.

[18] Nakahara T., Yamaguchi H., Uchida R. (2012). Effect of temperature on the stability of various peptidases during peptide-enriched soy sauce fermentation. J. Biosci. Bioeng..

[19] Vorobyev A., Hamidane H.B., Tsybin Y.O. (2009). Electron capture dissociation product ion abundances at the X amino acid in RAAAA-X-AAAAK peptides correlate with amino acid polarity and radical stability. J. Am. Soc. Mass Spectr..

[20] Wong F.C., Xiao J., Wang S., Ee K.Y., Chai T.T. (2020). Advances on the antioxidant peptides from edible plant sources. Trends Food Sci. Tech..

[21] Wu J., Sun B., Zhao M., Zheng F., Sun J., Sun X., Huang M. (2016). Discovery of a bioactive peptide, an angiotensin converting enzyme inhibitor in Chinese Baijiu. J. Chin. Inst. Food Sci. Technol..

[22] Ardö Y. (2006). Flavour formation by amino acid catabolism. Biotechnol. Adv..

[23] Li H., Wang C., Zhu L., Huang W., Yi B., Zhang L., Xu D. (2012). Variations of flavor substances in distillation process of Chinese Luzhou–flavor liquor. J. Food Process Eng..

[24] Lagesson-Andrasko L., Lagesson V., Andrasko J. (1998). The use of gas-phase UV spectra in the 168–330 nm wavelength region for analytical purposes. 1. qualitative measurements. Anal. Chem..

[25] Wu C., Liu J., Zhang X. (2018). Determination of organic acids in the root exudates of cr-hyperaccumulator *Leersia hexandra* Swartz using high performance liquid chromatography. Chin. J. Chromatogr..

[26] Stalikas C.D. (2007). Extraction, separation, and detection methods for phenolic acids and flavonoids. J. Sep. Sci..

[27] Wang R., Yang H.J., Yang X., Cao B.H. (2013). Four phenolic acids determined by an improved HPLC method with a programmed ultraviolet wavelength detection and their relationships with lignin content in 13 agricultural residue feeds. J. Sci. Food Agric..

[28] Moreno-Arribas M.V., Pueyo E., Polo M.C. (2002). Analytical methods for the characterization of proteins and peptides in wines. Anal. Chim. Acta.

[29] Grenier-Loustalot M.F., Lartigau C., Metras F., Grenier P. (1996). Mechanism of thermal polymerization of cyanate ester systems: Chromatographic and spectroscopic studies. J. Polym. Sci. Pol. Chem..

[30] He F., Duan J., Zhao J., Li H., Sun J., Huang M., Sun B. (2021). Different distillation stages Baijiu classification by temperature-programmed headspace-gas chromatography-ion mobility spectrometry and gas chromatography-olfactometry-mass spectrometry combined with chemometric strategies. Food Chem..

[31] Ding X., Wu C., Huang J., Zhou R. (2015). Changes in volatile compounds of Chinese Luzhou—Flavor liquor during the fermentation and distillation process. J. Food Sci..

[32] Ilczyszyn M., Chwaleba D., Mierzwicki K., Ilczyszyn M.M. (2008). Structural role of hydrogen bond networks in amino acid-acid systems.(I) The network with highly polarizable OHO hydrogen bonds in sarcosine-methanesulfonic acid (2:1) crystal. Chem. Phys..

