# Supplementary material for: Endogenous Peptides Identified in Soy Sauce Aroma Style Baijiu Which Interacts with the Main Flavor Compounds during the Distillation Process
Source: Foods. 2022 Oct 24;11(21):3339. doi: 10.3390/foods11213339 (PMC9657464; doi:10.3390/foods11213339)
Supplement: Supplementary file 1 [file foods-11-03339-s001.zip › foods-1897047-supplementary.pdf]

Supplementary Tables

Table S1. Composition of endogenous peptide in head liquor

Simple	Sequence	Length	Retention time	Mass	m/z	Sequence	Length	Retention time	Mass	m/z
Head liquor	EWTFAALAFLFKYLSRL	17	56.38	2075.12	1037.56	STLVGHDTFTK	11	21.56	1205.62	602.81
	HVLRLLRGVSMGAGVLP	17	56.60	1774.04	887.02	NQEVEEERLK	10	22.82	1273.63	636.81
	KTPSPLKPKRRRIIEN	16	56.52	1932.17	644.06	AEGALMAVGNAESR	14	18.91	1375.67	1375.67
	TPLQRKLSKLACLLFGI	17	56.20	1900.13	633.38	AVDAETAEK	9	6.26	933.45	466.73
	GQHSATTWSGQYVGGAEAR	20	56.11	1962.90	654.30	HDEAVDANSR	10	7.59	1113.49	556.75

Table S2. Composition of endogenous peptide in heart liquor

Simple	Sequence	Length	Retention time	Mass	m/z	Sequence	Length	Retention time	Mass	m/z
Heart liquor	TPLQRKLSKLACLLFGI	17	56.20	1900.13	471.29	HDEAVDANSR	10	6.37	1113.49	556.75
	NRARSKIV	8	10.15	942.57	573.68	PLLTHVSLVHTLLPVVLPFRKTT	23	53.56	2581.57	645.39
	NGLVQLGLRPTAKRKA	16	57.00	1721.04	633.38	TKWVTYTEPVQKL	13	22.09	1592.87	530.96
	RQVELAQR	8	9.61	999.57	499.78	HLQLALR	7	22.53	850.53	425.26
	RHVRPGTVALR	11	23.22	1261.76	420.59	TRPPREEELR	10	18.19	1282.70	427.57
	VVYESAVGNAESR	13	56.16	1380.68	1380.68	PPVQKLGTDPTPM	13	22.19	1380.73	690.37
	AVDAETAEK	9	7.82	933.45	466.73	VYTTKWPEVLR	11	29.96	1391.76	463.92
	PAHFKLPENFR	11	30.03	1355.73	451.91	YYQRLKRTPTVHLF	14	23.25	1822.03	455.51

Supplementary Figure

Figure S1. Endogenous peptides identified in head liquor by LC-MS/MS. (A) The corresponding liquid chromatogram. N: no peptides. P: peptides. (B) Total ion chromatogram. (C) Secondary mass spectrogram of the peptide corresponding to P_1 peak. (D) Secondary mass spectrogram of the peptide corresponding to P_2 peak.

Figure S2. Endogenous peptides identified in heart liquor by LC-MS/MS. (A) The corresponding liquid chromatogram. N: no peptides. P: peptides. (B) Total ion chromatogram. (C) Secondary mass spectrogram of the peptide corresponding to P_1 peak. (D) Secondary mass spectrogram of the peptide corresponding to P_2 peak. (E) Secondary mass spectrometry of the peptide corresponding to P_3 peak. (F) Secondary mass spectrometry of the peptide corresponding to P_4 peak.

Figure S3. Correlation analysis between the peptides and the main flavour substances in Baijiu samples from different distillation stages. (A) Spectra of the liquors from different distillation stages. (B) Correlation analysis among the characteristic peaks based on the maximum absorption value. (C) Concentration determination of total acid, total ester, total alcohol and the peptide. (D) Correlation analysis among the concentration of total acid, total ester, total alcohol and the peptides. Red represented positive, and blue represented negative. * $0.01< P \leq 0.05$, ** $P \leq 0.01$.

Figure S4. Molecular docking between the peptides and the major flavor substances. (A) STLVGHDTFTK in head liquor interacted with ethanol, ethyl acetate, lactic acid, and 2,3,5,6-tetramethylpyrazine. (B) TRPPREEELR in heart liquor interacted with ethanol, ethyl acetate, lactic acid, and 2,3,5,6-tetramethylpyrazine. (C) TRQVEERVW in tail liquor interacted with ethanol, ethyl acetate, lactic acid, and 2,3,5,6-tetramethylpyrazine.

Figure S1.

A

B

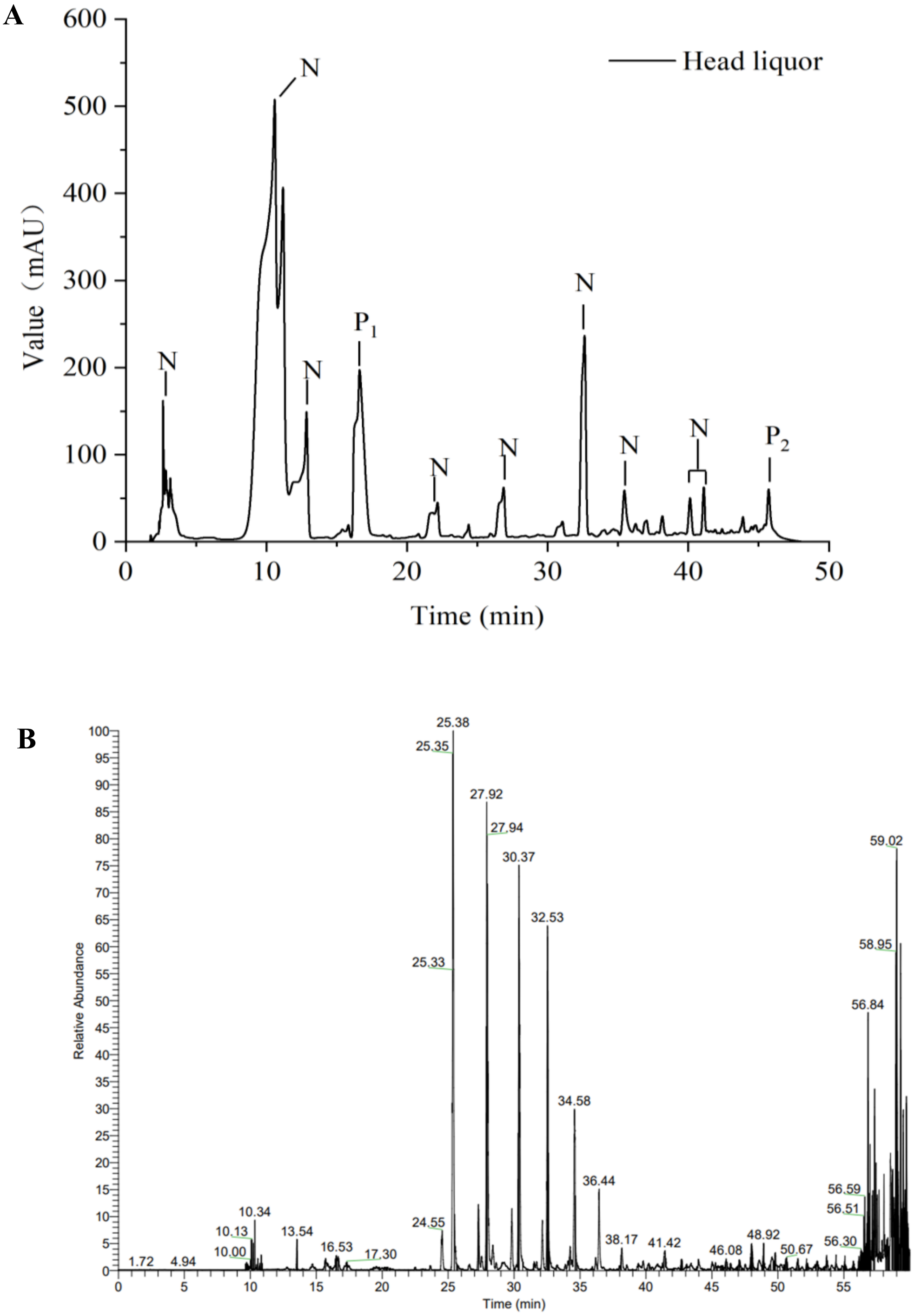
Head liquor
Value（mAU）
Time (min)
N
P1
P2
Relative Abundance
Time (min)
25.38
25.35
27.92
27.94
30.37
32.53
25.33
34.58
36.44
24.55
10.34
10.13
10.00
13.54
16.53
17.30
1.72
4.94
38.17
41.42
46.08
48.92
50.67
56.30
56.51
56.59
56.84
58.95
59.02

Figure S1 continued.

C

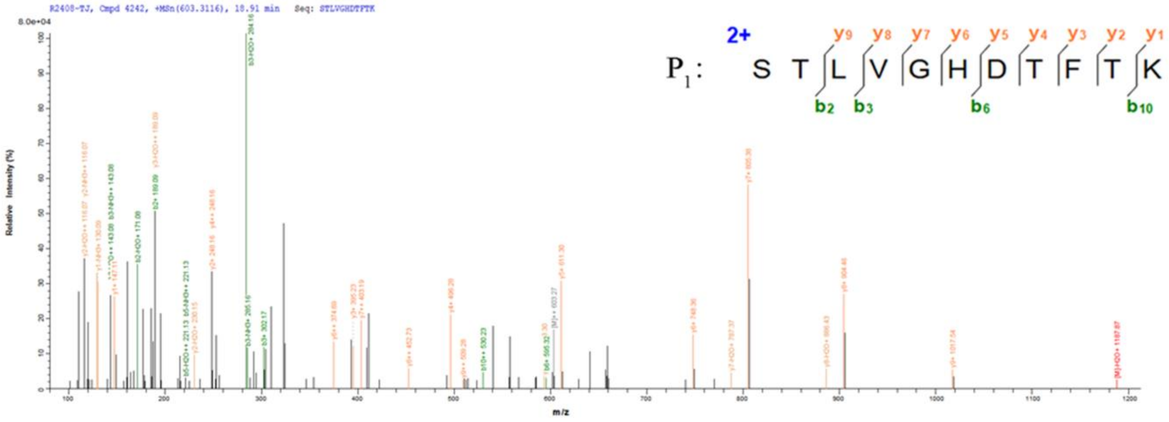

D

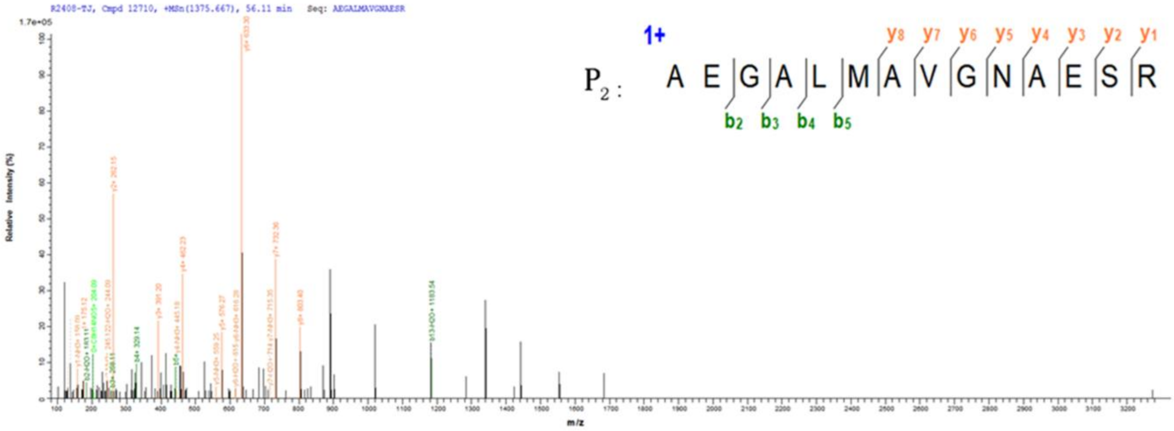

Figure S2.

A

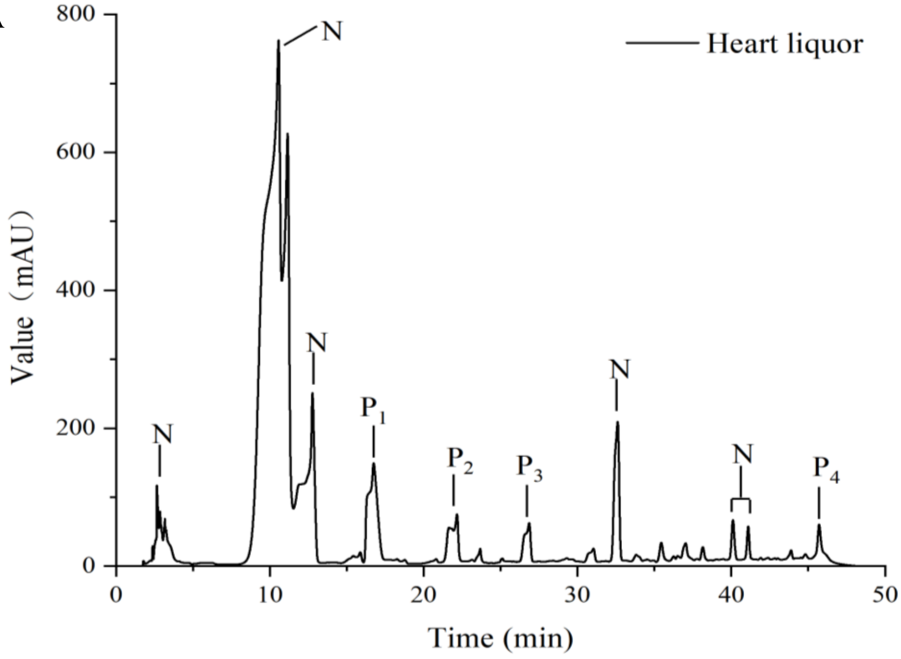
Heart liquor
Value（mAU）
Time (min)
N
P1
P2
P3
P4
0
200
400
600
800
10
20
30
40
50

B

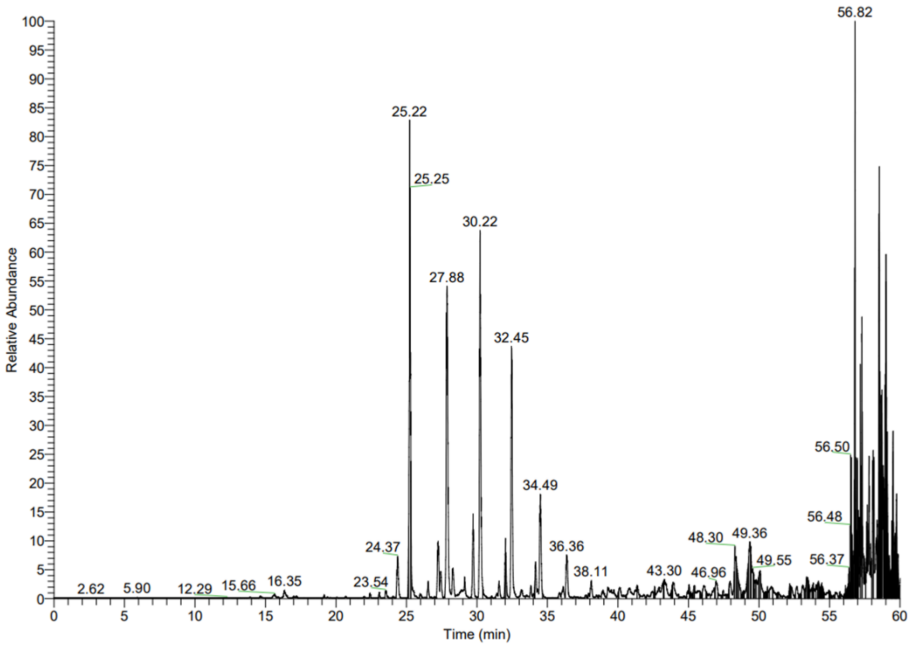
Relative Abundance
Time (min)
56.82
25.22
25.25
30.22
27.88
32.45
34.49
36.36
38.11
43.30
46.96
48.30
49.36
49.55
56.50
56.48
56.37
24.37
23.54
16.35
15.66
12.29
5.90
2.62

Figure S2 continued.

C

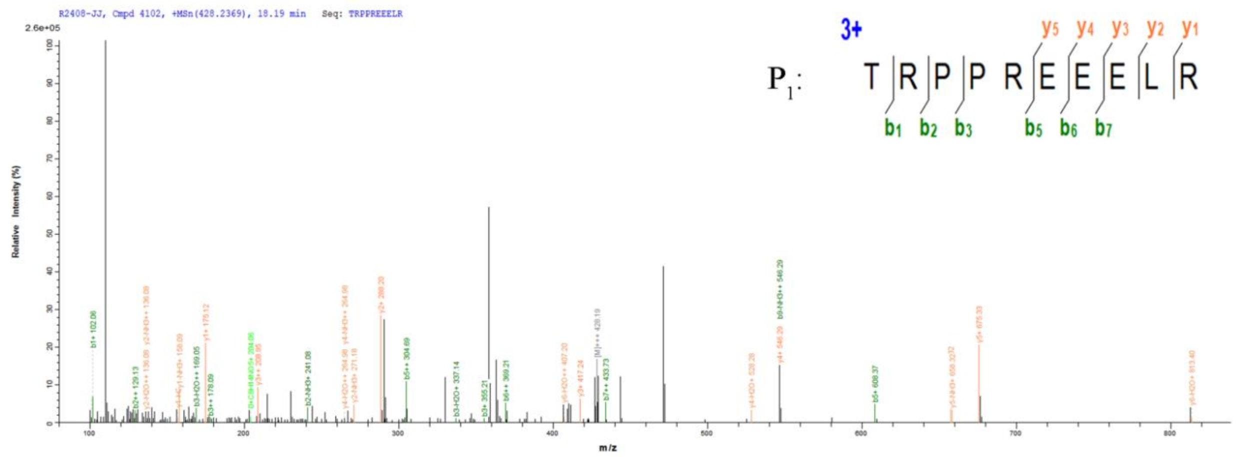

D

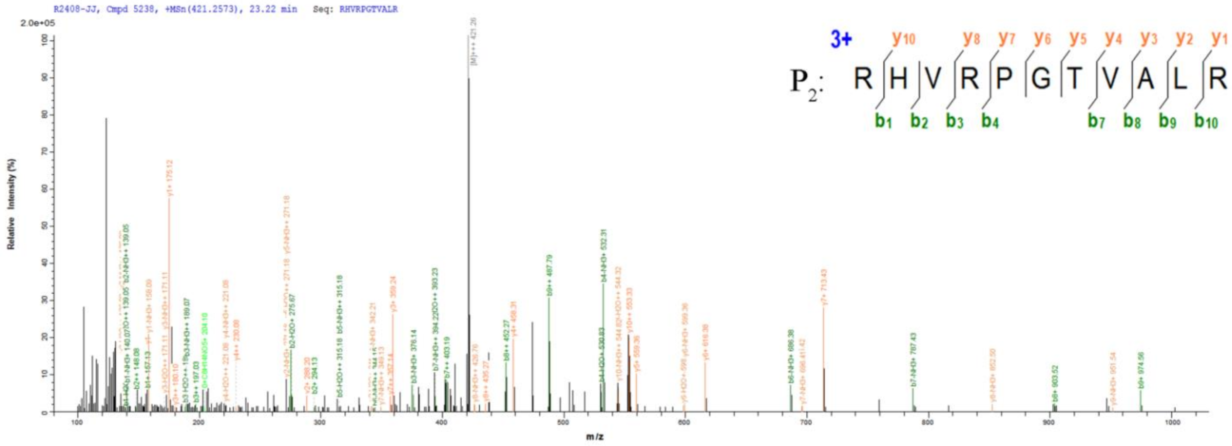

E

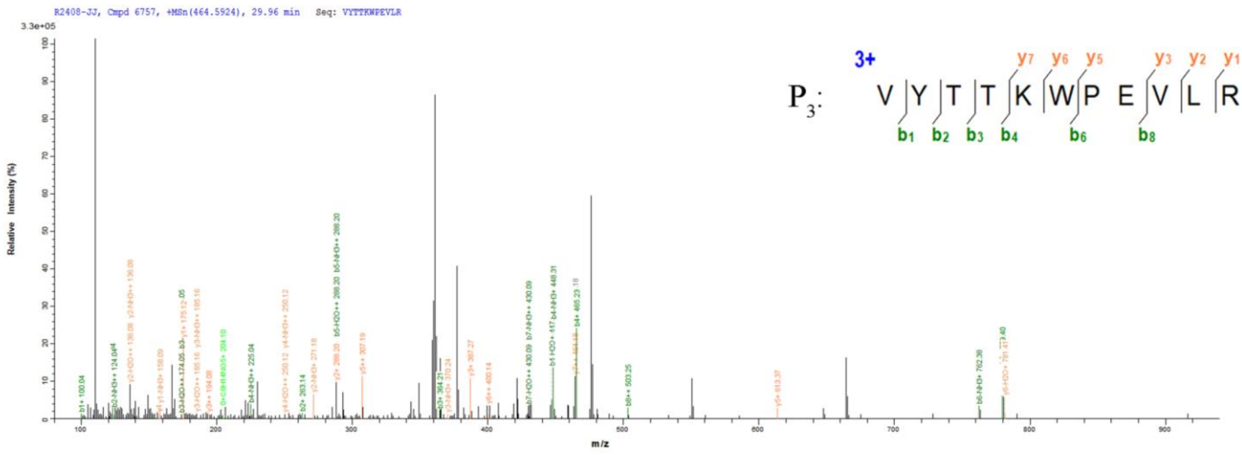

Figure S2 continued.

F

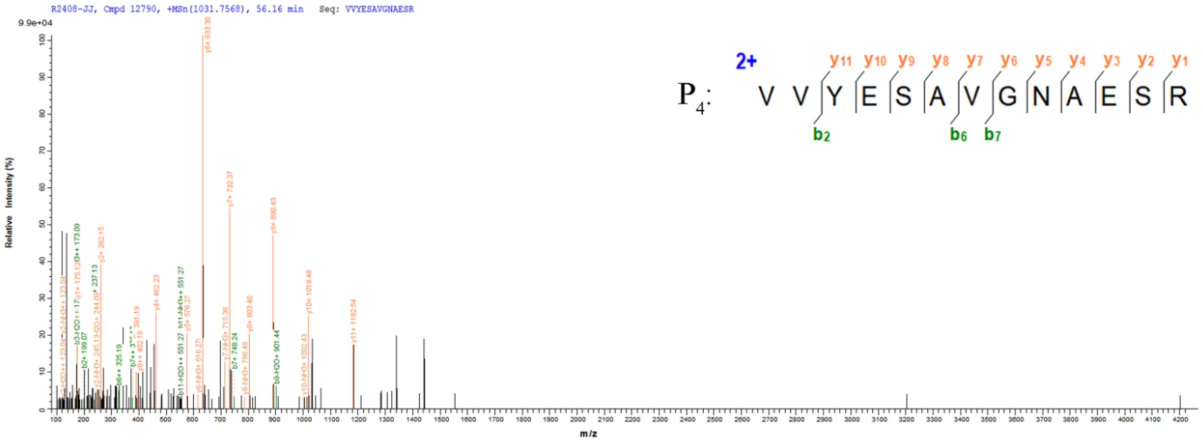

Figure S3.

A

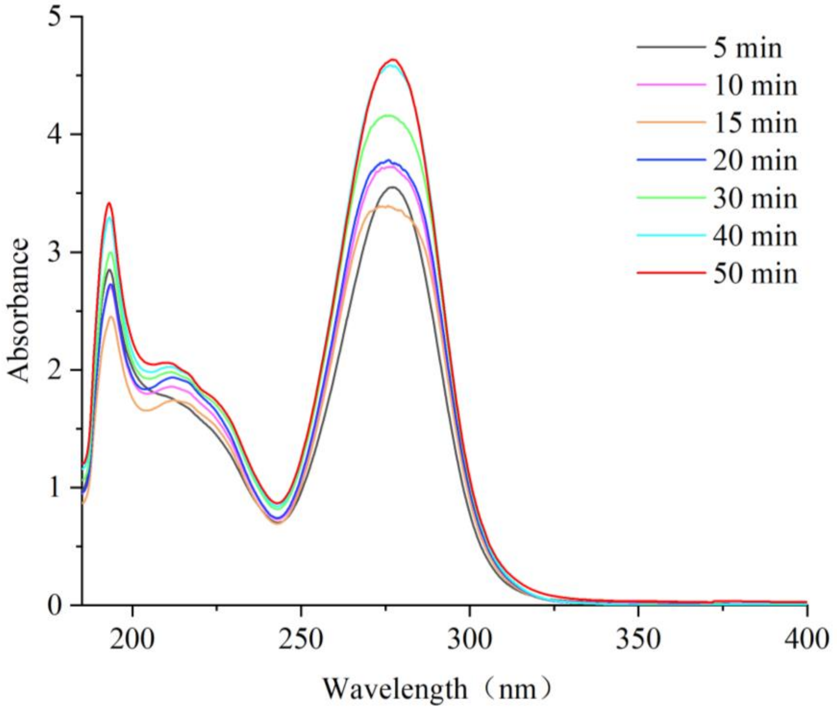
5
4
3
2
1
0
Absorbance
200
250
300
350
400
Wavelength （nm）
5 min
10 min
15 min
20 min
30 min
40 min
50 min

B

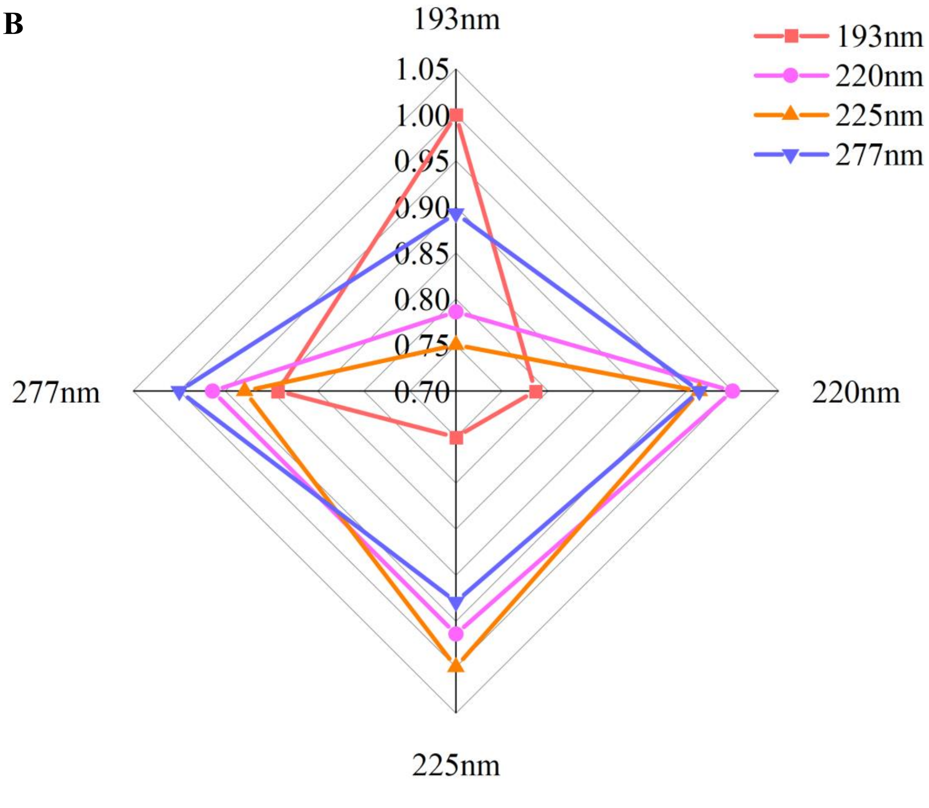
193nm
220nm
225nm
277nm
1.05
1.00
0.95
0.90
0.85
0.80
0.75
0.70
193nm
220nm
225nm
277nm

Figure S3 continued.

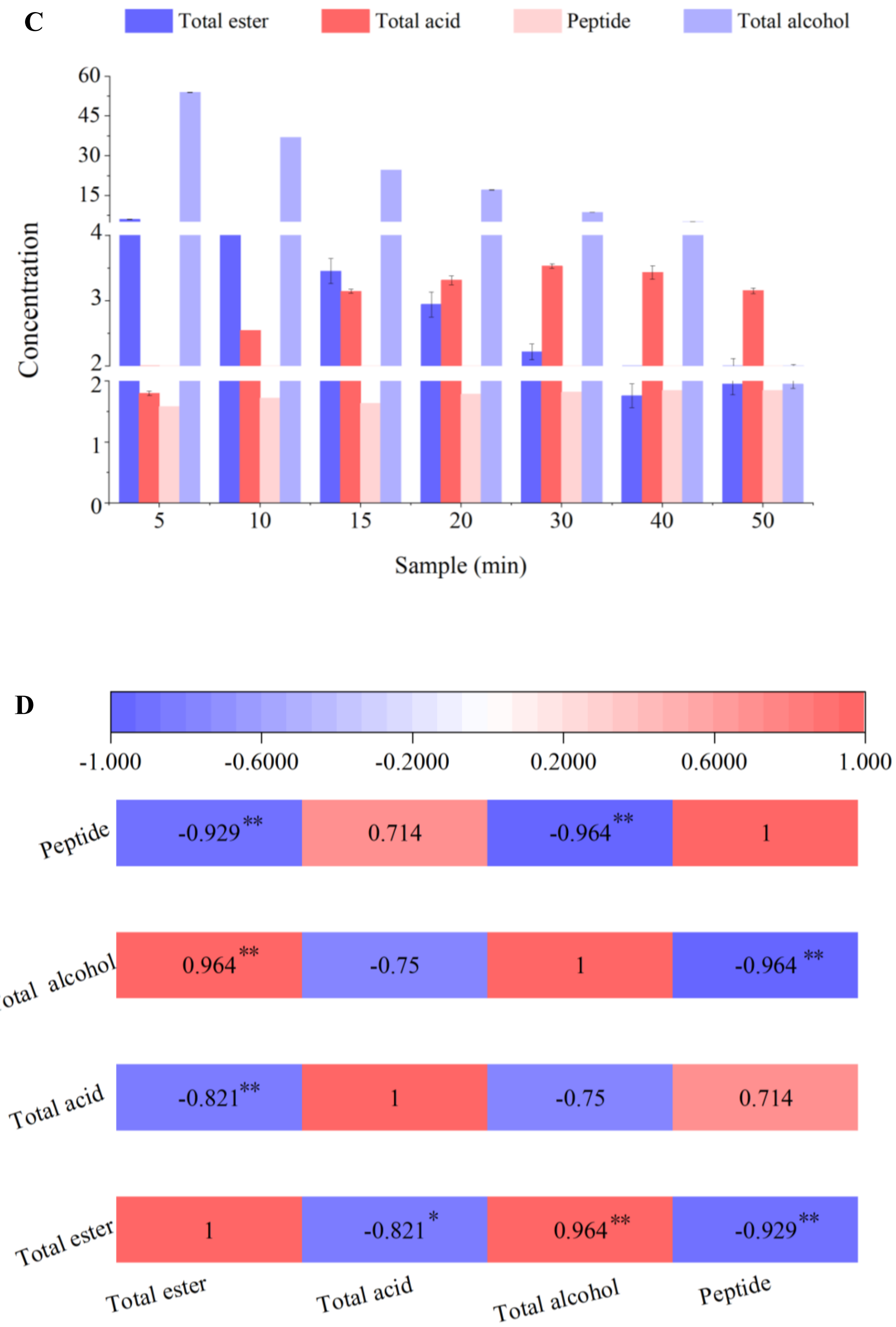

Figure S4.

A

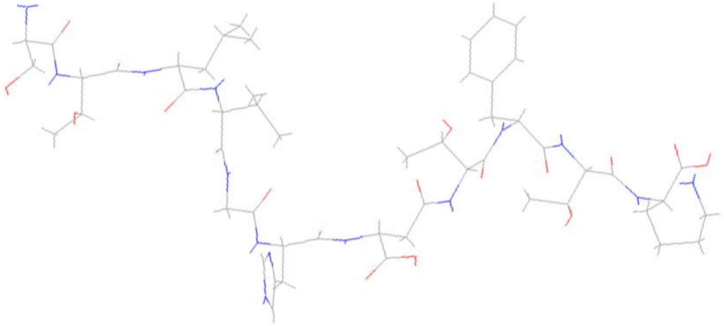

STLVGHDTFTK

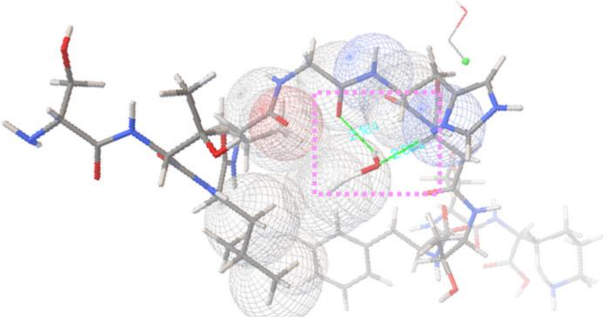

STLVGHDTFTK-Ethanol

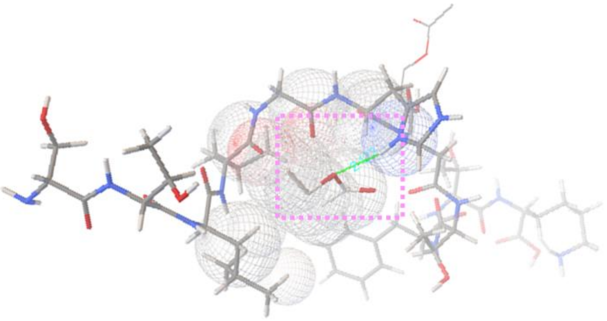

STLVGHDTFTK-Ethyl acetate

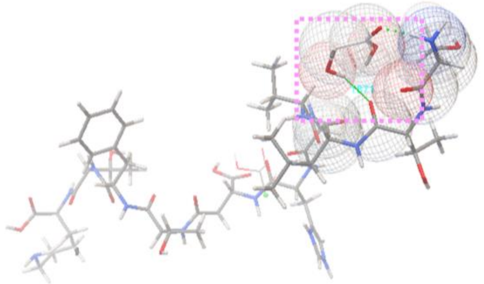

STLVGHDTFTK-Lactic acid

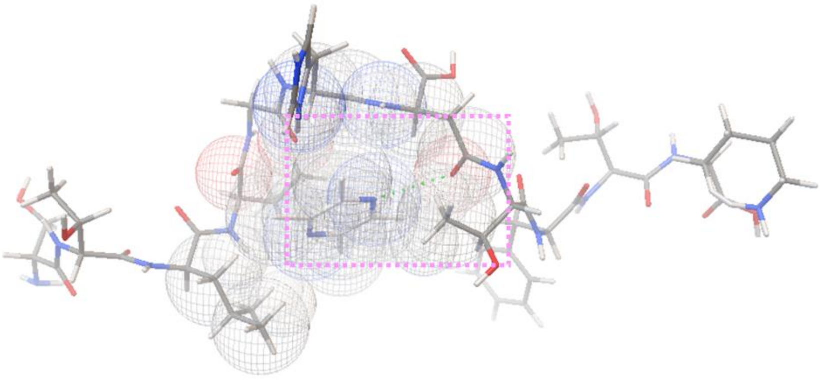

STLVGHDTFTK-2,3,5,6-Tetramethylpyrazine

Figure S4 continued.

B

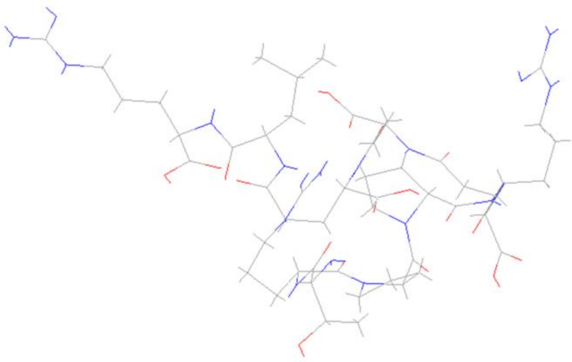

TRPPREEELR

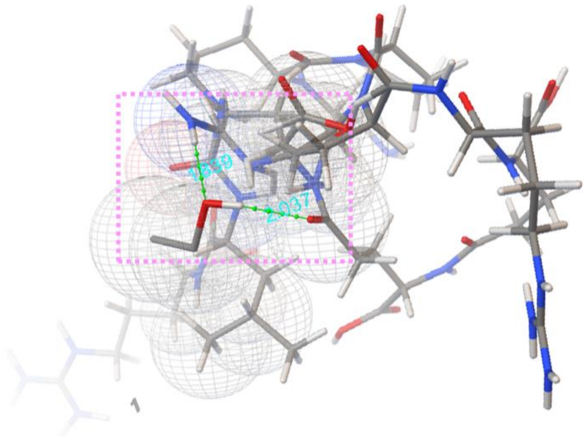

TRPPREEELR-Ethanol

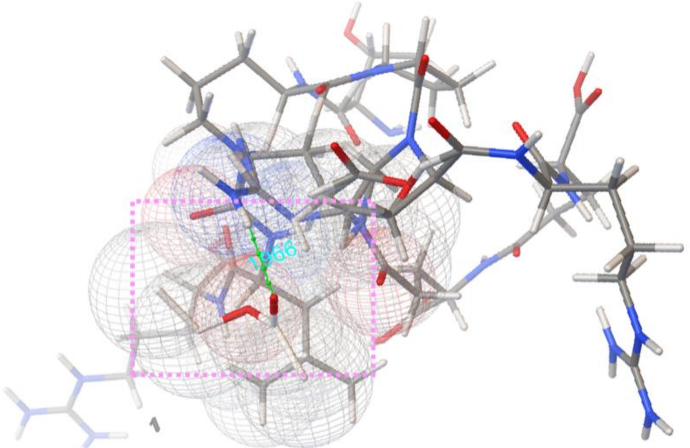

TRPPREEELR-Ethyl acetate

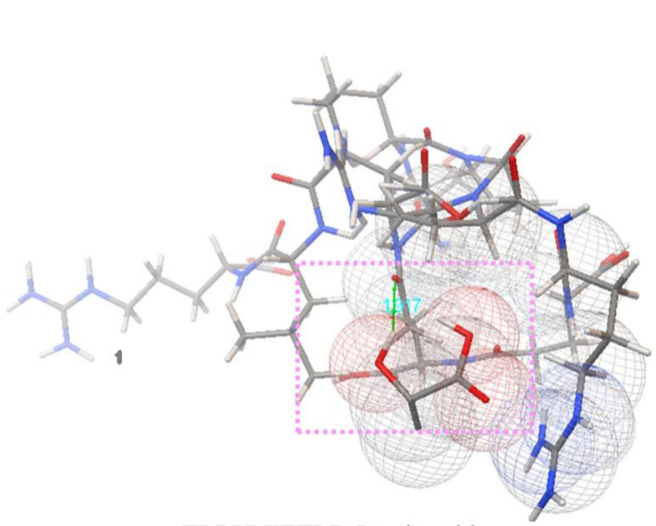

TRPPREEELR-Lactic acid

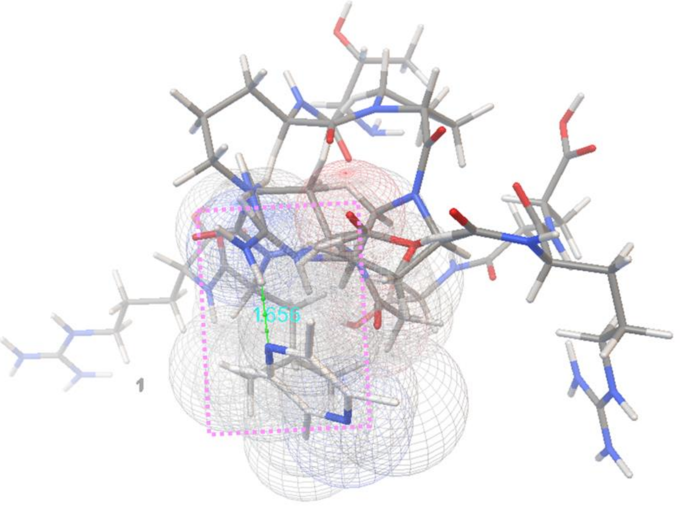

TRPPREEELR-2,3,5,6-Tetramethylpyrazine

Figure S4 continued.

C

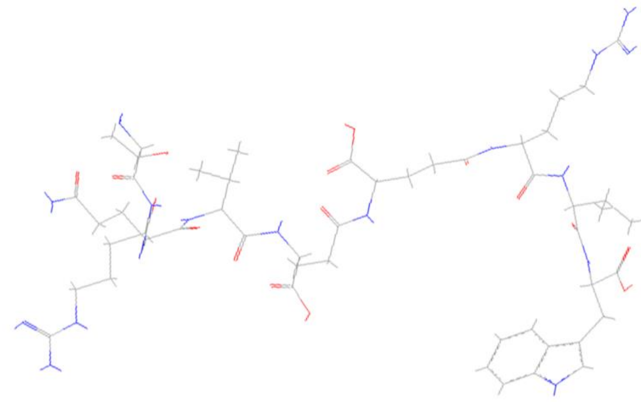

TRQVEERVW

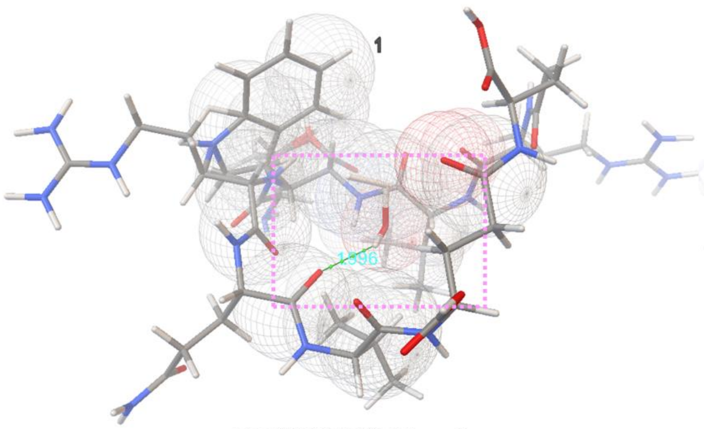

TRQVEERVW-Ethanol

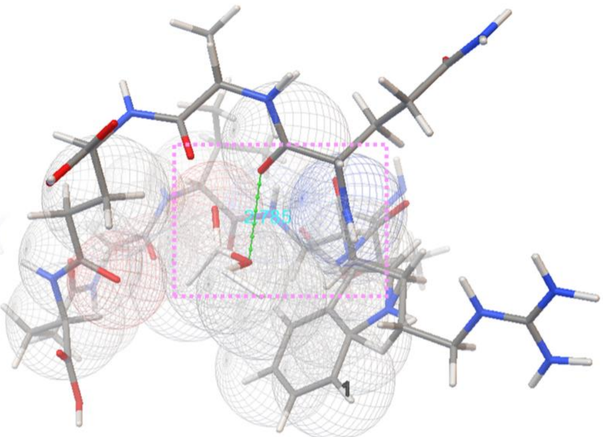

TRQVEERVW-Ethyl acetate

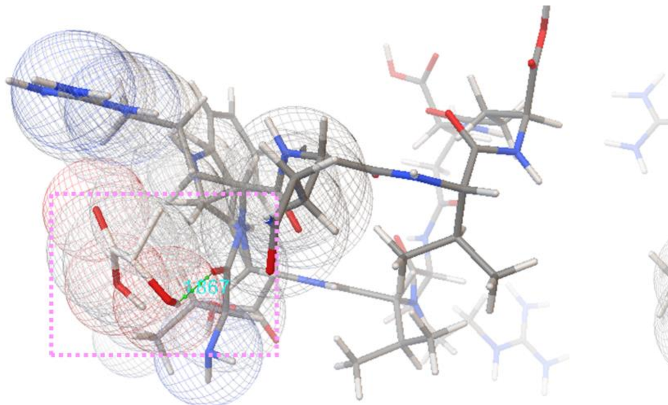

TRQVEERVW-Lactic acid

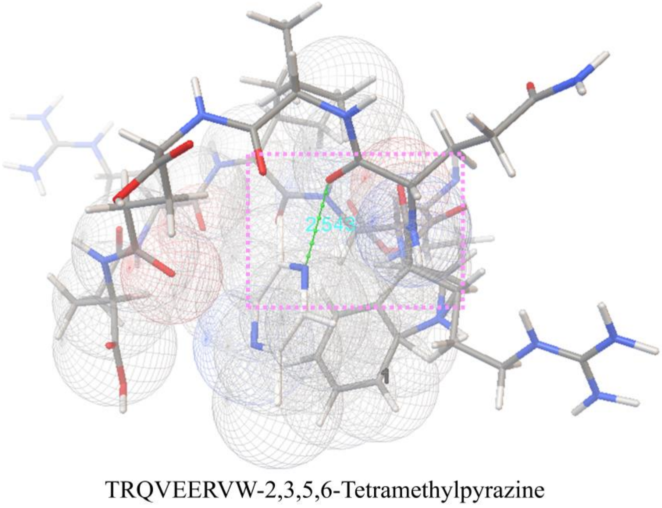

TRQVEERVW-2,3,5,6-Tetramethylpyrazine
